# Effects of LncRNA BC168687 siRNA on Diabetic Neuropathic Pain Mediated by P2X_7_ Receptor on SGCs in DRG of Rats

**DOI:** 10.1155/2017/7831251

**Published:** 2017-10-24

**Authors:** Chenglong Liu, Jia Tao, Hui Wu, Yixin Yang, Qiang Chen, Zeyu Deng, Jiandi Liu, Changshui Xu

**Affiliations:** ^1^Department of Physiology, Basic Medical College of Nanchang University, Nanchang, China; ^2^The Second Clinical Medical College of Nanchang University, Nanchang, China; ^3^The First Clinical Medical College of Nanchang University, Nanchang, China; ^4^Queen Mary College, Nanchang University, Nanchang, China

## Abstract

Diabetic neuropathic pain (DNP), one of the early symptoms of diabetic neuropathy, relates to metabolic disorders induced by high blood glucose, neurotrophic vascular ischemia and hypoxia, and autoimmune factors. This study was aimed at exploring the effects of long noncoding RNA (lncRNA) BC168687 siRNA on DNP mediated by P2X_7_ receptor on SGCs in DRG of rats. The mechanical withdrawal threshold (MWT) and thermal withdrawal latency (TWL) of rats, the expression levels of P2X_7_ mRNA and protein in the DRG, and nitric oxide (NO) in the serum were, respectively, detected in our study. Our experimental results showed that the level of BC168687 mRNA in DNP group was markedly higher than that of control group; the MWT and TWL of DNP + BC168687 si group were significantly increased, and the expression levels of P2X_7_ in DRG and the concentrations of NO in serum of DNP + BC168687 si group were decreased compared to those of the DNP group. In conclusion, lncRNA BC168687 may participate in the pathogenesis of DNP mediated by P2X_7_ receptor, which will provide a novel way for the study of the pathogenesis of diabetes mellitus complicated with neuropathic pain and its prevention and treatment.

## 1. Introduction

Long noncoding RNA (LncRNA), the cognition of which is still unclear, is a byproduct of RNA polymerase II transcription and has no biological function [1]. However, recent studies have shown that lncRNA is involved in many important regulatory processes, such as X-chromosome silencing, genomic imprinting, chromatin modification, transcriptional activation, transcriptional interference, and intranuclear transport, and these regulatory actions of lncRNA are beginning to arouse widespread attention [2, 3]. LncRNAs could produce a complex regulatory process by interacting with transcription factors, coactivators, and/or inhibitory factors to influence various aspects of gene transcription [4, 5]. Studies have demonstrated that knockout of mice lncRNAs can lead to functional abnormalities [6, 7]. Moreover, lncRNAs play an important role in the pathogenesis of neurological diseases [8].

Diabetic neuropathic pain (DNP), also known as painful diabetic peripheral neuropathy (PDN), is one of the most common types of neuropathic pain with quite complicated clinical manifestations [9] and could lead to spontaneous pain, hyperalgesia, and allodynia as well as other atypical paresthesia [10]. DNP is a kind of chronic pain whose mechanisms are complex and poorly understood, and effective therapies for DNP remain elusive [11], making DNP one of the major difficulties in the field of pain research for a long time [12]. DNP has severe effects on the quality of patients' life and could bring immense physical and mental suffering to the patients. Statistics indicate that the number of patients suffering from DNP accounts for 50%. Growing evidence supports that people with components of the metabolic syndrome, including prediabetes, are also likely to suffer from neuropathy [13].

Adenosine triphosphate (ATP) is involved in the transmission of analgesia, and there are evidences that extracellular ATP plays important roles via activation of purinoceptors in the physiological and pathological processes of neuropathic pain [14–16]. Purinoceptors 2 (P2 receptors) can be divided into ligand-gated ion channels receptor P2X (P2X_1–7_) and G-protein linked receptor P2Y [17, 18]. DRG transmit sensory signals from the peripheral nerve to the spinal cord [19]. P2X receptors and acid-sensing ion channels (ASICs) are both ligand-gated ion channels, and they are different in primary structures as well as responding to ligands (P2X receptors are gated by extracellular ATP, while ASICs are activated by extracellular acidification) [20]. The ATP-gated P2X_7_ receptor expressed on satellite glial cells (SGCs) has previously been shown to play a central role in the pain transmission and the occurrence of neuropathic pain in diabetes mellitus patients [21]. Studies have indicated that, compared with wild-type mice, sensitivities to mechanical pain and thermal pain are decreased significantly in P2X_7_ receptor-knockout mice [22]. Conversely, the activation of P2X_7_ receptor can lead to the release of inflammatory factors, thus initiating chronic pain such as neuropathic pain [23, 24]. Furthermore, antagonists of the P2X_7_ receptor could inhibit the mechanical pain and thermal pain of neuropathic pain rats [25, 26].

LncRNA BC168687 is one of the lncRNAs (https://www.ncbi.nlm.nih.gov/nuccore/BC168687) [27]; however, unfortunately, the function of lncRNA BC168687 is unclear at present. Our study showed that the level of lncRNA BC168687 was augmented in the DRG of DNP rats, suggesting that lncRNA BC168687 might be related to the transmission of analgesia signaling; thus inhibiting lncRNA functions* in vivo* may be a hopeful therapeutic strategy to treat some diseases [6–8]. This research explored the effects of lncRNA BC168687 siRNA on P2X_7_ receptor mediated neuropathic pain in diabetic model rats through the system-level tests, which will provide a novel way for the study of the pathogenesis of diabetes mellitus complicated with neuropathic pain and its prevention and treatment.

## 2. Materials and Methods

### 2.1. Animal Model and Groups

Healthy male Sprague-Dawley (SD) rats (weight 180–220 g) were provided by the Laboratory Animal Science Department of Nanchang University. All the rats were kept under constant conditions (22 ± 2°C and 12 h light/dark cycle) with free access to food and water according to the Care and Use of Animals guidelines set by the Ethical Committee of Nanchang University. The rats were fed with high-sugar and high-fat diet (formula quality ratio: conventional feed 66.5%, cholesterol 2.5%, sodium cholate 1%, lard 10%, and sucrose 20%; the materials above were mixed with water and kneaded into a ball and then placed into an air dry oven with constant temperature) for four weeks, and after that the rats were injected intraperitoneally with a dosage of 30 mg/kg streptozotocin (STZ) solution. After one week, the mechanical withdrawal threshold (MWT) and fasting plasma glucose (FPG) of the rats were measured to evaluate the establishment of DNP models, and the blood was obtained from the tail vein. DNP of rats was defined as FPG > 16.7 mmol/L and MWT < 15 g after 6 weeks.

The rats were randomly divided into control group, DNP model group (DNP), DNP treated with BC168687 siRNA group (DNP + BC168687 si), DNP treated with the scrambled siRNA group (DNP + NC si), and DNP treated with empty vector group (DNP + vector), and each group contained 6 rats. The target sequences of BC168687 siRNA were sense 5′-GACGGUUGAUACUGACUCUTT-3′ and antisense 5′-AGAGUCAGUAUCAACCGUCTT-3′. The BC168687 siRNA and negative control (NC siRNA) were provided by Novobio Company (Shanghai, China). The Entranster™-*in vivo* transfection reagents were obtained from the Engreen Company (Engreen Biosystem Co, Ltd.). According to the instructions of the reagent, the DNP + BC168687 si group and DNP + NC si group rats were injected with 25 *μ*l of transfection complexes comprising BC168687 siRNA or NC siRNA through intrathecal injection, and 25 *μ*l of normal saline was injected into control and DNP group rats. The behavior of the rats was tested on the 1st, 3rd, and 5th days after injection. After the measurement of blood glucose and behavior on the fifth day, the rats were anesthetized by intraperitoneal injection with 50 mg/kg sodium pentobarbital and sacrificed to collect the DRG.

### 2.2. Measurement of the Mechanical Withdrawal Threshold (MWT)

Mechanical hyperalgesia was measured by noxious-pressure stimulation. Unrestrained rats were placed into a transparent plastic chamber (22 × 12 × 22 cm) standing on a stainless steel mesh floor (1 × 1 cm grid). All rats were adapted to the new environment before the trials were started. Room temperature was maintained at 20–25°C with environment kept quiet. Withdrawal responses to mechanical stimulation were determined using BME-404 Electrical Mechanical Analgesia Tester (BME-404, Tianjin) through the grids in the floor to an area adjoining to the paw. Random bending force was exerted through the filaments to different parts of the rat mid plantar glabrous skin for 1 min. The average strength of six selected peak values was designated as the pain threshold.

### 2.3. Measurement of the Thermal Withdrawal Latency (TWL)

Noxious heat stimulation was implemented by the BME-410C Full-Automatic Plantar Analgesia Tester (BME-410C, Tianjin), and Hargreaves' test was used for determining hyperalgesia. The rats were placed on a glass plate within a transparent bottomless plastic chamber (22 × 12 × 22 cm) and were allowed to habituate for 30 min. TWL was considered as an index of the nociceptive threshold. At the beginning of trails, the plantar surface of the rats' paws was exposed to a beam of radiant heat. Once a rat lifted its paw, the light beam was automatically turned off immediately, and the time from the start of the light beam to the raising of the foot was recorded to be specified as the paw thermal withdrawal latency. The hind paws were tested alternately at intervals of 5 min. The cut-off time for heat stimuli was 30 s.

### 2.4. Quantitative Real-Time PCR

The total amount of RNA was extracted with Total RNA Isolation Kit (Beijing Tiangen Biotech Co.), and the RNA was reverse-transcribed using a RevertAid™ First-Strand cDNA Synthesis Kit (Fermentas, Glen Bernie, MD, USA) according to the manufacturer's instructions. After first-strand cDNA synthesis, quantitative PCR amplification was conducted using the specific primers designed with the Primer Express 3.0 software (Applied Biosystems), and the sequences were as follows: *β*-actin, sense 5′-CCTAAGGCCAACCGTGAAAAGA-3′, antisense 5′-GGTACGACCAGAGGCATACA-3′; P2X_7_, sense 5′-GATGGATGGACCCACAAAGT-3′, antisense 5′-GCTTCTTTCCCTTCCTCAGC-3′. SYBR® Green quantitative PCR was performed using an ABI PRISM® 7500 Sequence Detection System (Applied Biosystems, Inc., Foster City, CA, USA). The thermal cycling parameters were as follows: 95°C for 30 s to activate DNA polymerase, followed by 40 cycles of amplification at 95°C for 5 s and 60°C for 30 s [28]. The melting curve was used to determine the amplification specificity, and the results were analysed by the software with the ABI7500 PCR instrument. The average threshold cycle (CT) value for P2X_7_ minus the average value for *β*-actin was ΔCT value (ΔCT = CT target − CT reference). And ΔΔCT value was calculated as follows: ΔΔCT = ΔCT test sample  −  ΔCT calibrator sample. The relative quantity (RQ) of P2X_7_ expression was calculated using the following equation: RQ = 2^−ΔΔCT^.

### 2.5. Reverse Transcription-PCR

The total amount of RNA was extracted with Total RNA Isolation Kit (Beijing Tiangen Biotech Co.) and PCR amplification was implemented after RT reaction. The upstream and downstream primer sequences were as follows: lncRNA BC168687, sense 5′-CACCACCTGGATGACATGCT-3′, antisense 5′-GGTGGCATCCTTTGACTGGA-3′, with the product size being 102 bp; P2X_7_, sense 5′-GATGGATGGACCCACAAAGT-3′, antisense 5′-GCTTCTTTCCCTTCCTCAGC-3′, with the product size being 115 bp. As an internal control, *β*-actin was also amplified using specific primers (forward and reverse primer sequence genes were 5′-CCTAAGGCCAACCGTGAAAAGA-3′ and 5′-GGTACGACCAGAGGCATACA-3′, resp.) with the product size being 111 bp. After reverse transcription at 37°C for 60 min, PCR amplification was performed as follows: RTase inactivation at 94°C for 3 min, followed by 31 cycles of denaturation at 94°C for 45 s and annealing at 60°C for 30 s, and ultimately extension at 72°C for 45 s. The spot density scanning value of the objective electrophoresis bands was detected by using the multifunctional gel imaging system (BIO-RAD, USA). Image LabC gel imaging system software (BIO-RAD, USA) was used to read the band densities. Acting as an internal control, *β*-actin was used to normalize each band density to show the relative level of lncRNA BC168687 and P2X_7_ mRNA.

### 2.6. Immunofluorescence

Immunofluorescence was performed to measure the coexpression quantities of P2X_7_ and glial fibrillary acidic protein (GFAP) in the DRG of DNP rats. The DRG of the rats were removed and fixed in 4% PFA for 2 h at room temperature. The DRG or sections were washed three times with 0.1 M PBS before the implementation of the following steps. DRG were incubated in 20% sucrose in PBS overnight, and after that, they were cut into 12 *μ*m thick sections using a cryostat and mounted onto the slides. Then the DRG were blocked and permeabilized in PBS containing 3% BSA and 0.3% Triton X-100 for 30 min at room temperature. The sections were incubated with primary antibodies against GFAP (chicken anti-GFAP, Abcam) and P2X_7_ (rabbit anti-P2X_7_, Abcam) 1 : 100 diluted in PBS containing 1% BSA overnight at 4°C, after which they were incubated with the secondary antibodies [goat anti-rabbit TRITC (Jackson ImmunoResearch Inc., West Grove, PA, USA) and goat anti-chicken FITC (Beijing Zhongshan Biotech Co.)] 1 : 200 diluted in PBS for 45 min at 37°C. Finally, coverslips were mounted on slides and images were taken with a fluorescence microscope (Olympus, Tokyo, Japan).

### 2.7. Western Blotting

Total protein was extracted for western blotting analysis. The DRG tissues were homogenized in lysis buffer by mechanical disruption and incubated on ice for 50 min. After that, the lysates were centrifuged at 12,000 ×g and 4°C for 10 min. The supernatants were harvested to measure the protein concentrations using a bicinchoninic acid assay reagent kit and then preserved at −20°C for later use. After being diluted with the same amount of sample buffer (250 mmol/L Tris-Cl, 200 mmol/L dithiothreitol, 10% SDS, 0.5% bromophenol blue, and 50% glycerol), the supernatants were heated to 95°C for 10 min for denaturation. Supernatant samples containing 20 *μ*g proteins were separated by 10% SDS-polyacrylamide gels and transferred to polyvinylidene fluoride (PVDF) membranes. The PVDF membranes were blocked in 5% skim milk dissolved in buffer [10 mmol/L Tris-HCl (pH 8.0), 150 mmol/L NaCl, and 0.05% Tween-20] for 1 h at room temperature and then incubated with the primary antibodies (rabbit anti-P2X_7_, 1 : 1000, Abcam; mouse anti-*β*-actin, Sigma-Aldrich) overnight at 4°C, followed by incubation with an HRP-conjugated secondary antibody (1 : 5000, Beijing Zhongshan Biotech Co.) at room temperature. The band densities were determined using Image J Software and normalized to each *β*-actin internal control.

### 2.8. Determination of Nitric Oxide (NO)

NO in the serum was detected by using NO assay kit (Nanjing Jiancheng Bioengineering Institute, China). All the operations in the experiment were carried out in accordance with the specification. The OD values were read by the multifunctional microplate reader at 550 nm (PerkinElmer, USA), and then the result was calculated according to the formula given by the specification.

### 2.9. Statistical Analysis

Significant differences were evaluated using analysis of variance (ANOVA), followed by* Fisher's* Least Significant Difference (LSD) test for multiple comparisons. The statistical analyses were performed using SPSS 21.0 (IBM, USA), and all of the histograms were made in GraphPad Prism 5.0 (GraphPad Software Inc., USA). All of the data were expressed as mean ± SD, and significance was considered as *P* < 0.05.

## 3. Results

### 3.1. The Level of LncRNA BC168687 in DRG Increased in DNP Model Rats

**Figure 1:**
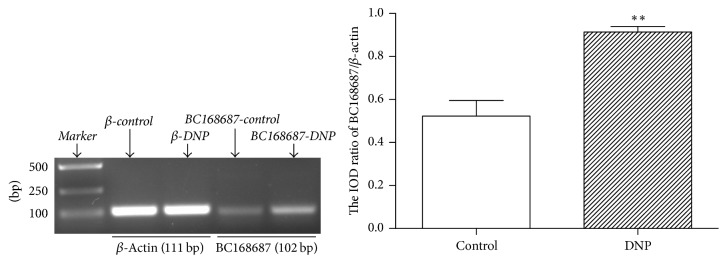
The level of LncRNA BC168687 in DRG increased in DNP rats by reverse transcription-PCR. The level of lncRNA BC168687 in DNP group was significantly higher than that in the control group. *n* = 6, ^*∗∗*^*P* < 0.01 versus control group.

Studies had showed that dysregulation of lncRNAs was associated with nervous system diseases. Reverse transcription-PCR was conducted to evaluate the level of lncRNA BC168687 in DRG. Our experimental results revealed that the level of lncRNA BC168687 in DRG of the DNP group was significantly higher than that of the control group by reverse transcription-PCR (Figure 1), indicating that lncRNA BC168687 was involved in the initiation of DNP in rats.

### 3.2. BC168687 siRNA Decreased the Mechanical Hyperalgesia and Thermal Hyperalgesia of DNP Rats

**Figure 2:**
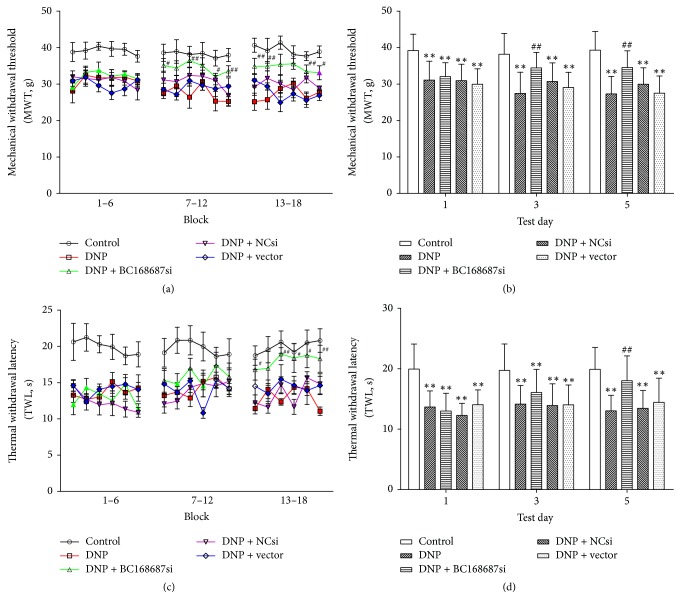
BC168687 siRNA increased the MWT and TWL of DNP rats. A 3-day protocol consisting of six blocks was performed on each group (consisting of six rats) on the 1st, 3rd, and 5th days after intrathecal injection with BC168687 siRNA. Three days after inoculation, block analysis of MWT (a, b) and TWL (c, d) indicated lower threshold of DNP rats compared to the control rats and higher threshold of DNP + BC168687 si rats compared to the DNP rats. Symbols (a, c) filled with color indicate significance; unfilled symbols represent no significance versus control group; ^*∗∗*^*P* < 0.01, versus control group; ^#^*P* < 0.05, ^##^*P* < 0.01, DNP + BC168687si group versus DNP group.

Mechanical hyperalgesia and thermal hyperalgesia will be enhanced during DNP state. The effects of the BC168687 siRNA on mechanical hyperalgesia and thermal hyperalgesia were tested by using BME-404 Electrical Mechanical Analgesia Tester and BME-410C Full-Automatic Plantar Analgesia Tester, respectively. A two-way ANOVA was performed to examine the effect of treatment and time on MWT and TWL. Simple main effects analysis showed that the effect of treatment was statistically significant (*F*_4,525_ = 87.878, *P* < 0.01, for the MWT, Figure 2(a); *F*_4,525_ = 68.837, *P* < 0.01, for the TWL, Figure 2(c)). There was an interaction between time factors and treatment factors in the MWT (*F*_8,525_ = 3.050, *P* < 0.01) and the TWL (*F*_8,525_ = 4.209, *P* < 0.01); after intrathecal injection with BC168687 siRNA, the MWT and TWL of each group increased on the 1st, 3rd, and 5th days (as shown in Figure 2), indicating that BC168687 siRNA contributed to alleviating DNP. In terms of the ANOVA results, the MWT of each group was statistically significant (*F*_4,535_ = 58.007, *P* < 0.01, Figure 2(b)), and similar results were also obtained about the TWL (*F*_4,535_ = 65.501, *P* < 0.01, Figure 2(d)). Followed by LSD test for comparative analysis between two groups, at the 1st, 3rd, and 5th days, the MWT and TWL of the DNP group, the DNP + NC si group, and the DNP + vector group were significantly lower than those in control group, while, compared to the DNP group, the MWT and TWL were increased in the DNP + BC168687 si group (*P* < 0.01). For example, on the 5th day, the MWT (34.54 ± 4.59 g, *n* = 36) and the TWL (18.03 ± 4.09 s, *n* = 36) of DNP + BC168687 si rats were significantly (*P* < 0.01) higher than those of DNP rats (27.30 ± 4.81 g, *n* = 36, for the MWT; 13.02 ± 2.57 s, *n* = 36, for the TWL), while there was no significant difference among the DNP, DNP + NC si, and DNP + vector group (*P* > 0.05). The data revealed that MWT and TWL in DNP model rats were increased after injection with BC168687 siRNA.

### 3.3. BC168687 siRNA Reduced P2X_7_ mRNA Expression of DRG in DNP Rats

**Figure 3:**
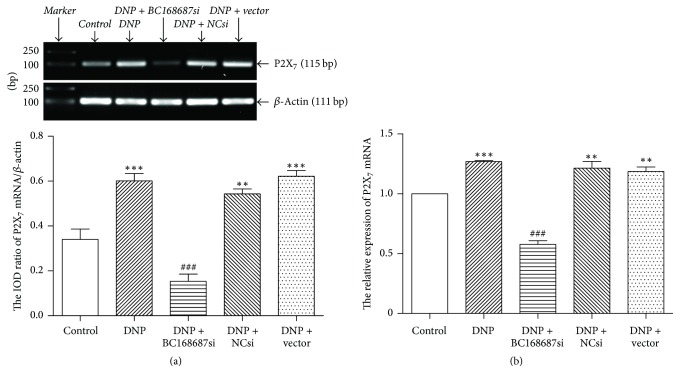
BC168687 siRNA reduced P2X_7_ mRNA expression of DRG in DNP rats. (a) The relative expressions of P2X_7_ mRNA in DRG by reverse transcription-PCR; (b) the relative expressions of P2X_7_ mRNA in DRG by qPCR. The expression level of the P2X_7_ mRNA in the DNP group was higher than that in the control group. The expression level of the P2X_7_ mRNA in the BC168687 si group was lower than that in the DNP group. *n* = 6, ^*∗∗*^*P* < 0.01, ^*∗∗∗*^*P* < 0.001 versus control group; ^###^*P* < 0.001 versus DNP group.

The expression level of P2X_7_ mRNA in DRG was detected by reverse transcription-PCR (Figure 3(a)). The results showed that the relative expressions of P2X_7_ mRNA in control, DNP, DNP + BC168687 si, DNP + NC si, and DNP + vector groups were 0.34 ± 0.08, 0.60 ± 0.05, 0.15 ± 0.05, 0.54 ± 0.03, and 0.62 ± 0.04, respectively, and the variance was statistically significant (*F*_4,10_ = 37.53, *P* < 0.001). Compared to the control group, the expression level of P2X_7_ mRNA in the DNP group increased significantly. The expression level of P2X_7_ mRNA in the DNP + BC168687 si group was lower than that in the DNP group (*P* < 0.001). No significant difference appeared among the DNP, the DNP + NC si, and the DNP + vector groups (*P* > 0.05). In order to ensure the accuracy of the results, we repeated the experiment by qPCR (Figure 3(b)), and the results were similar to RT-PCR. The relative expressions of P2X_7_ mRNA by qPCR in control, DNP, DNP + BC168687 si, DNP + NC si, and DNP + vector groups were 1.00 ± 0.00, 1.26 ± 0.01, 0.57 ± 0.05, 1.21 ± 0.09, and 1.18 ± 0.06, respectively; and the variance analysis was statistically significant (*F*_4,10_ = 72.67, *P* < 0.01). Based on the results obtained, we concluded that BC168687 siRNA could influence the upregulation of P2X_7_ mRNA in the DNP rats.

### 3.4. The Coexpression of P2X_7_ and GFAP in DRG Based on Immunofluorescence

**Figure 4:**
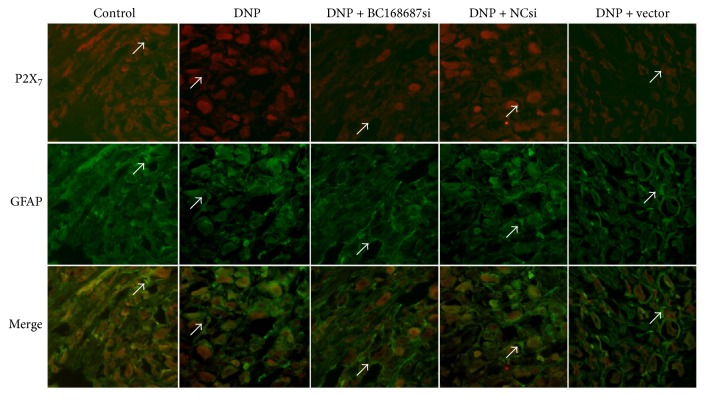
The coexpression quantities of P2X_7_ and GFAP were increased in the DRG of DNP rats. The coexpression of P2X_7_ receptor and GFAP in the DNP group was higher than that in the control group, whereas the coexpression value in the DNP + BC168687 si group was lower than that in DNP group. The green signal represents GFAP staining with FITC (fluorescein isothiocyanate), and the red signal indicates P2X_7_ staining with TRITC (tetraethyl rhodamine isothiocyanate). Merge represents the P2X_7_ and GFAP double staining image. The arrows indicate the positive cells in DRG. Scale bar: 20 *μ*m.

GFAP is the marker of satellite glial cells (SGCs). Immunofluorescence technique was used to detect the coexpression of P2X_7_ receptor and GFAP in DRG. The fact that the expression of GFAP in the DRG was upregulated revealed that SGCs were activated in the condition of nervous injury stimulus [29]. What have been shown in Figure 4 are the coexpression quantities of the P2X_7_ receptors and GFAP in the five groups detected on the 5th experimental day based on the colocalization of P2X_7_ and GFAP on SGCs in DRG. When compared to control group, the coexpression quantities of the P2X_7_ receptors and GFAP increased in the DNP group and the coexpression quantities of the DNP group were higher than that of the DNP + BC168687 si group. No apparent difference was observed among the DNP, the DNP + NC si, and the DNP + vector groups. Therefore, we inferred that BC168687 siRNA may reduce the upregulation of the P2X_7_ receptors which were related to the activation of SGC in the DRG.

### 3.5. BC168687 siRNA Downregulated the P2X_7_ Protein Expression of DRG in DNP Rats

**Figure 5:**
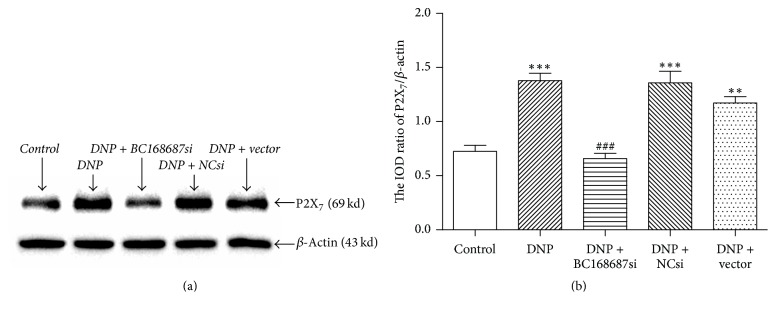
BC168687 siRNA downregulated the P2X_7_ protein expression of DRG in DNP rats. (a) Representative SDS-PAGE electrophoresis of western blotting for P2X_7_ receptor (69 kd) and an internal control of the same sample's *β*-actin (43 kd). (b) Relative expression level of P2X_7_ receptor protein in each group. The expression of P2X_7_ protein in DNP group showed a significant increase compared to the control group. The DNP + BC168687 si group showed a decrease in the expression level of P2X_7_ protein compared to the DNP group. *n* = 6, ^*∗∗*^*P* < 0.05, ^*∗∗∗*^*P* < 0.001 versus the control group; ^###^*P* < 0.001 versus the DNP group.

The P2X_7_ protein expression was detected by western blotting (Figure 5). The result showed that the relative expressions of P2X_7_ protein of the control, the DNP, the DNP + BC168687 si, the DNP + NC si, and the DNP + vector groups were 0.72 ± 0.09, 1.37 ± 0.11, 0.65 ± 0.08, 1.35 ± 0.18, and 1.17 ± 0.10, respectively, and the variance analysis was statistically significant (*F*_4,10_ = 24.11, *P* < 0.001). The expression of P2X_7_ protein in the DNP group was higher than that in the control group (*P* < 0.001), while, compared to the DNP group, the expression level of the P2X_7_ protein in the DNP + BC168687 si group decreased markedly (*P* < 0.001), but no significant differences were observed among the DNP, the DNP + NC si, and the DNP + vector groups (*P* > 0.05). Therefore, BC168687 siRNA may influence the upregulation of P2X_7_ receptor in the DNP rats.

### 3.6. Effects of BC168687 siRNA on NO Level in the Serum of DNP Rats

**Figure 6:**
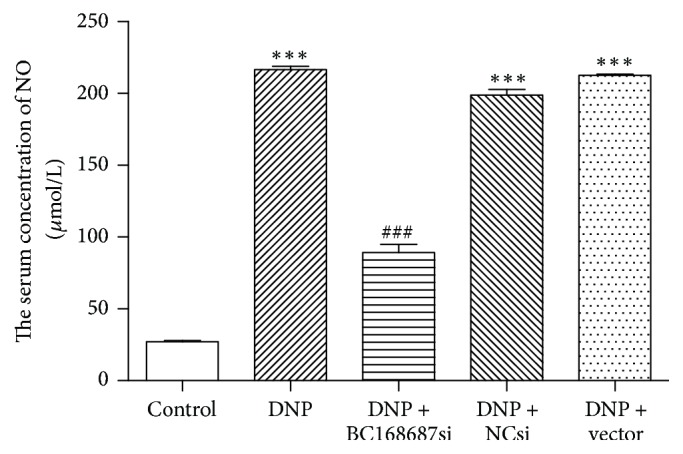
BC168687 siRNA reduced NO level in the serum of DNP rats. The level of NO in DNP group showed a significant increase compared to the control group. The DNP + BC168687 si group showed a decrease in the level of NO compared to the DNP group. *n* = 6, ^*∗∗∗*^*P* < 0.001 versus the control group; ^###^*P* < 0.001 versus the DNP group.

NO, as a kind of oxidative injury factor released from SGCs, was considered to initiate and maintain neuropathic pain. Results showed that the NO concentrations (*μ*mol/L) of serum in the control, DNP, DNP + BC168687 si, DNP + NC si, and DNP + vector groups were 27.21 ± 1.40, 216.51 ± 4.20, 89.29 ± 9.54, 198.77 ± 6.88, and 212.53 ± 1.40, respectively, and the variance analysis was statistically significant (*F*_4,10_ = 689.80, *P* < 0.001). The NO level in the DNP group was considerably higher than that in the control group (*P* < 0.001), and the NO level in the DNP + BC168687 si group was lower than that in the DNP group (*P* < 0.001), while there was no statistical difference among the DNP group, the DNP + NC si group, and the DNP + vector group (*P* > 0.05) (Figure 6).

## 4. Discussion

DNP is a common neurological complication in diabetic patients, but the current treatment of it is far from satisfactory. LncRNAs have been proved to perform important cellular functions through many* in vitro* researches and considered to contribute to the pathogenesis of neurological diseases [7, 30]. Recent experiments suggested directions for the development of disease therapies targeting lncRNAs [31–33]. Through the reverse transcription-PCR experiment discussed above, we found that the level of lncRNA BC168687 in the DRG of the DNP group was significantly higher than that in the control group. In combination with the fact that the dysregulation of lncRNAs was associated with numerous diseases, including nervous system diseases [34], we inferred that the upregulation of lncRNA BC168687 in DRG may be associated with the pathological changes that contribute to DNP.

Diabetic neuropathy is the general term of a variety of diseases caused by diabetes mellitus in the nervous system [35]. As one of the most common, complex, and serious complications of patients with diabetes, diabetic neuropathy is a kind of nervous system damage caused by the state of chronic hyperglycemia and other various pathophysiological changes induced by diabetes, and any part of the systemic peripheral nervous system (including sensory nerve, motor nerve, and autonomic nerve) can be affected [36, 37]. Clinically, diabetic neuropathy has been manifested as spontaneous pain, hyperalgesia, and allodynia or even developed to diabetic foot ulcers or required amputation, which has serious impacts on the life quality of patients [38, 39]. As our experimental results showed, the MWT and TWL of DNP rats were lower than those of the control rats, while, compared to the DNP rats, the MWT and TWL of DNP rats treated with the BC168687 siRNA* in vivo* were increased. In addition, a further test showed that no significant difference of MWT or TWL was observed among the DNP group, the DNP + NC si group, and the DNP + vector group, indicating that it was not the scrambled siRNA or empty vector treatment but the BC168687 siRNA treatment that exerted significant effect on the MWT and TWL. Therefore, suppressing lncRNA BC168687 may alleviate pain in DNP rats. However, the underlying mechanism of the effect of the BC168687 siRNA on the neuropathic pain diabetic rats is still poorly understood and requires further investigation.

DRG accepted pain signals from peripheral nerve and transmitted them to the central nervous system [40, 41]. Several P2X receptor subtypes, such as P2X_3_ and P2X_7_, have been proved to have distinct effect on the pathogenesis of central pain [41]. P2X_3_ receptor is mainly located in primary sensory neurons and can elevate the nociception by directly sensitizing pain fibers, thus being involved in pathological pain [42]. However, P2X_7_ receptor expressed on the SGCs in DRG was regarded as a more recent participant in the field of purinergic signaling in chronic pain [42]. Previous researches have demonstrated that P2X_7_ exerts various effects in neuropathic and inflammatory pain due to its role in the inflammasome activation and maturation of IL-1*β* [41, 43]. We observed that the P2X_7_ mRNA and protein expression levels in the DRG of DNP rats were significantly increased compared with DNP + BC168687 si group rats, while there was no apparent difference among the DNP group, the DNP + NC si group, and the DNP + vector group, indicating that BC168687 siRNA could inhibit the expression of P2X_7_ receptor to influence the pathological process of DNP.

It has been generally assumed that the somata of neurons in primary sensory ganglia are tightly enwrapped by satellite glial cells (SGCs) [44, 45]. The SGCs in DRG mainly expressed P2X_7_ receptor [15, 46] and could communicate with neurons via signaling molecules. Our experiment revealed that P2X_7_ receptor was coexpressed with GFAP on SGCs based on immunofluorescence, and the coexpression levels of P2X_7_ and GFAP in DRG were upregulated in the DNP rats compared with the control rats. As is known to all, GFAP is an SGC marker [47], and SGCs can be activated in the condition of nervous system pathological injuries [45, 48]. In DNP rats, BC168687 siRNA suppressed the upregulated coexpression values of P2X_7_ and GFAP on SGCs in DRG, indicating that the BC168687 siRNA may reduce the upregulation of the P2X_7_ receptor related to SGCs activation in DRG.

Studies demonstrated that there existed interaction between neurons and SGCs in the DRG [45, 48, 49]. ATP released from nerve terminals could activate the P2X_7_ receptor on the SGCs in DRG, thus stimulating SGCs to release oxidative injury factors, such as NO that was involved in the initiation and maintenance of neuropathic pain [50]. In addition, the excitability of neurons in DRG and their sensitivity to noxious stimulation could be strengthened with NO existing [51]. Our results showed that the level of NO in serum of the DNP group was higher than that of the control group, while, in BC168687 si group, the level of NO in serum was significantly decreased. Based on these results, we inferred that BC168687 siRNA may suppress P2X_7_ activation on SGCs to inhibit the upregulation of NO, thus decreasing the nociceptive behaviors of DNP rats.

## 5. Conclusions

In summary, our experimental results displayed that the expression of P2X_7_ receptors in the DRG of DNP rats was reduced after BC168687 siRNA intrathecal injection. Furthermore, the BC168687 siRNA may reduce the release of NO and the activation of SGCs, thus restraining the DRG neurons from being excited and relieving the pain behaviors of DNP rats. These findings manifested that the P2X_7_ receptors on SGCs in DRG played an important role in mediating DNP and highlighted the potential of lncRNA BC168687 as a novel therapeutic target for DNP.
